# Complex Adsorption Pattern Formation in Drying BSA–NaCl Droplets: Experimental Study

**DOI:** 10.3390/ijms27115060

**Published:** 2026-06-03

**Authors:** Polina Borshchegovskaya, Violetta Kim, Ulyana Bliznyuk, Alexander Chernyaev, Victoria Ipatova, Maria Toropygina, Alexander Nikitchenko, Aleksandr Kozlov, Igor Rodin, Elena Kozlova

**Affiliations:** 1 Department of Physics, Lomonosov Moscow State University, GSP-1, 1-2 Leninskiye Gory, 119991 Moscow, Russia; ivantcova.vs20@physics.msu.ru (V.K.); uabliznyuk@gmail.com (U.B.); a.p.chernyaev@yandex.ru (A.C.); nikitchenko.ad15@physics.msu.ru (A.N.); 2 Skobeltsyn Institute of Nuclear Physics, Lomonosov Moscow State University, GSP-1, 1-2 Leninskiye Gory, 119991 Moscow, Russia; ipatova.vs15@physics.msu.ru; 3 Department of Medical and Biological Physics, Sechenov First Moscow State Medical University, 8/2 Trubetskaya Str., 119048 Moscow, Russia; tim.mmit@yandex.ru (M.T.); fillnoise@mail.ru (A.K.); waterlake@mail.ru (E.K.); 4 Department of Chemistry, Lomonosov Moscow State University, GSP-1, 1-3 Leninskiye Gory, 119991 Moscow, Russia; igorrodin@yandex.ru; 5 F.F. Erisman Institute of Public Health of Sechenov University, 8-2 Trubetskaya Str., 119991 Moscow, Russia

**Keywords:** bovine serum albumin, drying droplets, proteins in aqueous-salt solutions, salt crystal, self-organization of aggregate-crystal structures

## Abstract

The interaction between proteins and salts in aqueous solutions represents a compelling scientific problem in both biophysical and medical research. One manifestation of the interaction in the bovine serum albumin (BSA)–NaCl–H_2_O system is the formation of ordered patterns upon droplet drying. In our experiments, the topographic features of the deposited particles as well as their elemental composition were studied using optical microscopy and scanning electron microscopy (SEM). In this work, we experimentally investigated the stepwise change in the characteristic structures of the precipitate with an increase in the BSA concentration from 0.005 to 35 mg/mL. The formation of discrete BSA–NaCl ring deposits near the droplet edge and around crystallization centers in the interior at BSA concentrations of 0.05–1 mg/mL proved particularly interesting. We demonstrated the sequence of ring structure formation: the process primarily begins with the formation of BSA aggregates arranged in sectors around the circumference, which in turn serve as nucleation sites for NaCl crystallization. We propose a qualitative conceptual–phenomenological interpretation of the observed experimental effects. Concentration-dependent patterns in the emergence and development of other patterns (such as spikes, fractal structures, and chrysanthemum-like formations) were established. These results expand our understanding of protein behavior in aqueous-salt solutions. This can be used in medical diagnostics as biomarkers. The characteristic patterns presented in the work can serve as a useful experimental basis for further studies of the impact of physicochemical factors on proteins and other biopolymers.

## 1. Introduction

The study of self-organization processes leading to the formation of ordered structures in drying colloidal droplets of biological fluids is of scientific interest for understanding synergistic interactions in multicomponent biological systems.

Numerous experiments have demonstrated that the structure of patterns formed during the drying of droplets of biopolymer–salt solutions (ranging from coffee-ring structure to dendritic patterns) correlates with various system parameters, including the chemical composition of the solution [1,2,3,4,5,6], the concentration of components in the solution [2,3,6,7,8,9,10], external conditions such as humidity [2,3,10] and temperature [3,7,11,12], as well as the droplet size [13].

An array of experimental data has been accumulated on patterns that emerged from saline solutions of various components (fibrinogen [1,14], lysozyme [3,8,15], collagen [1], albumin [1,3,5,9,12,15,16], sodium polystyrene sulfonate [2], etc.). The types of low-molecular salts (LiCl, NaCl, KCl, RbCl NaI, etc.) are also varied in experiments [2,12,14,17,18,19]. The most convenient model system for studying the emergence of self-organizing ordered structures is bovine serum albumin (BSA) in a NaCl solution [3,5,9,10,12,19,20].

To visualize the nano- and microstructure of sediments formed after drying of polymer–salt solutions, the following microscopy methods are used: optical microscopy [1,2,5,9,10,13,14,15,21,22], atomic force microscopy (AFM) [4,5,13,14,19,22], and electron microscopy [1,7,16].

The concentration of BSA plays a critical role in the formation of ordered structures in BSA + NaCl mixtures. In many studies, characteristic patterns have been examined at protein concentrations comparable to or exceeding those of the salts present. For instance, with 0.9% NaCl, BSA concentrations on the order of 10 mg/mL have been investigated [7,17] as well as local concentrations reaching 90 mg/mL [5] and higher [10,18,20]. Several studies suggest that phase separation occurs during droplet drying, resulting in both BSA aggregates and NaCl crystals appearing in the sediment [1,8,9,22].

The studies present data on the formation of a broad spectrum of protein-crystalline structures arising during the drying of BSA + salt solutions over a range of concentrations [5,8,10,14,18]. As a result of molecular interactions, various crystal morphologies are formed: single-needle polycrystals [10] at concentrations of 10 mg/mL; within the protein concentration range from 10 mg/mL, dendritic [10,12,19] and fractal structures [5,10,17,18,19,22], as well as other aggregates [7], are observed. Starting from 50 mg/mL, the protein may limit crystal growth, leading to a fine-grained pattern [23]; multi-needle ‘snowflake’-like structures appear at 110 mg/mL [18]. At these concentrations, a ‘coffee-ring’ pattern forms at the droplet edge [3,8,10,12,23], or an ‘eye-like’ pattern emerges at 160 mg/mL [10]. Altering the ratio of biopolymer to salt concentrations can significantly modify the morphology of the resulting patterns [5,8,10,14,24].

To describe the mechanisms of pattern formation in drying droplets of biological fluids, models have been proposed that account for solvent evaporation dynamics, including radial capillary flows [18,25,26] directed toward the droplet edge with a pinned contact line [25,26,27], which explain the formation mechanism of the ‘coffee-ring’ structure [25,26,27,28]. As evaporation proceeds and the salt concentration in polymer-containing solutions (including BSA) increases, the ionic strength of the medium rises, leading to charge screening of protein molecules and thereby promoting their aggregation [8,14,18,20,23,29]. Particular attention is given to the role of proteins in initiating salt crystallization upon reaching a critical concentration [30].

Further understanding of the physicochemical mechanisms underlying the formation of ordered deposits during the drying of multicomponent biological systems will enable the application of the drying sessile droplet method for diagnostic purposes [18,31], the development of pharmaceutical formulations [4,7], and the design of functional materials [1,25].

In studies [6,21], the authors investigate the dynamics of optical and mechanical properties of droplets of biological fluids (blood serum, plasma, saliva, urine, etc.) during drying, revealing morphological features under normal conditions and in various diseases or physiological states [6,18,21]. Upon reaching certain concentrations, changes in the external environment, and interactions with surrounding molecules, dissolved proteins form aggregates [20,30], which can contribute to pathological changes in biological tissues [29]. Currently, research increasingly notes a variety of diseases associated with proteinopathies [32] and deviations in substance concentrations from physiological norms [21]. Understanding the structural features of protein aggregates arising at different concentrations may aid in the development of medical diagnostic methods [18,21,30,31,33]. Despite the large number of studies, experimental investigation and comprehension of the role of self-organization processes in the formation of ordered structures in drying droplets remain an important and active research challenge [23,26,33].

In the human body, albumin concentration spans a wide range: 30–55 mg/mL in blood [23,24,34], 4–16 mg/mL in interstitial fluid [34], 0.1–0.3 mg/mL in cerebrospinal fluid [24], and down to 0.03 mg/mL in urine [35]. Deviations in these concentrations in either direction may indicate possible complications [21,36].

The present study aims to investigate the kinetics of formation of adsorptive structures ensembles of BSA and salts at physiological BSA concentrations ranging from 0.005 to 35 mg/mL during droplet drying. In this work, we demonstrated how BSA deposit patterns in physiological solution were changed during droplet evaporation and identified the specific patterns that emerge at different BSA concentrations. Quantitative assessments of parameters and characteristics of the resulting patterns are provided. Phase separation effects—between BSA aggregates and salt crystals—were observed and recorded. The application of optical and electron microscopy in our study allowed us to obtain images of the patterns at nano- and micrometer scales across different time points recorded in video sequences as well as to measure elemental composition at various locations within the deposits and correlate it with the topography of the corresponding regions. These data contribute to a deeper understanding of the synergistic interplay of heterogeneous processes.

## 2. Results

### 2.1. Study Stages

**Figure 1:**
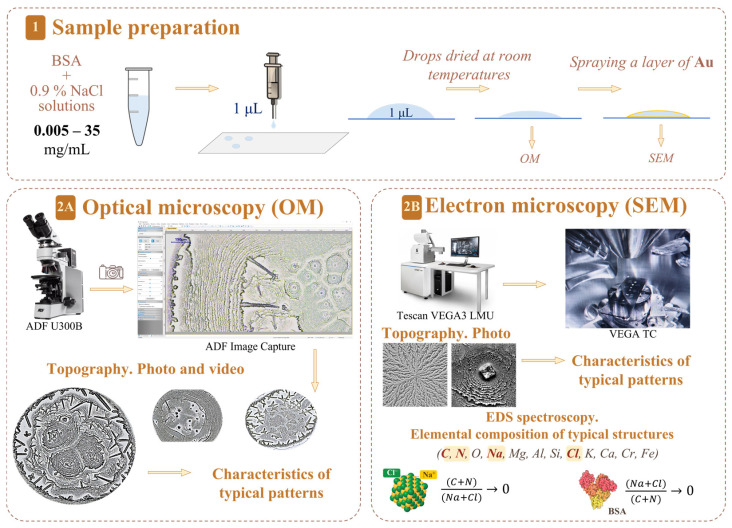
Stages of experimental study: (**1**) preparation of BSA solution in various concentrations for dried droplet samples on a microscope slide for further analysis under an optical microscope (**2A**) and an electron microscope (**2B**) with preliminary spraying of a thin layer of conductive material (gold).

This study examined the topography of adsorption structures in a protein–salt solution ${BSA+NaCl+H}_{2}O$ during drop drying. BSA concentrations ranged from C_0.005_ = 0.005 mg/mL to C_35_ = 35 mg/mL, diluted in 0.9% NaCl. Figure 1 shows the two stages of the study, details of each of which are given in the Materials and Methods section.

In the *first stage*, samples of dried droplets from a bovine serum albumin (BSA) solution were prepared. To do this, a dry weight of 350 mg of BSA was diluted in 10 mL of physiological solution (0.9% NaCl), and then, this solution was diluted to working BSA concentrations C_0.005_–C_35_. A 1 µL drop of each working solution concentration was applied to a microscope slide and dried at room temperature (22 °C, 295 K). Samples prepared for analysis under an electron microscope were additionally sprayed with a thin layer of conductive material (gold).

During the *second stage*, a topographical study of adsorbed structures was conducted by photos and videos with optical microscopy (2A) and of adsorption structures on nano- and micrometer scales using scanning electron microscopy (2B). Energy-dispersive X-ray spectroscopy (EDS) was applied to determine the elemental composition of typical BSA –NaCl in the dried droplets.

Based on the topographical analysis, the spatial and temporal characteristics of the resulting deposition structures were investigated.

### 2.2. Formation of Various Adsorption Patterns During Drying of a Drop in a Wide Range of BSA Concentrations from 0.05 mg/mL to 35 mg/mL in 0.9% NaCl

**Figure 2:**
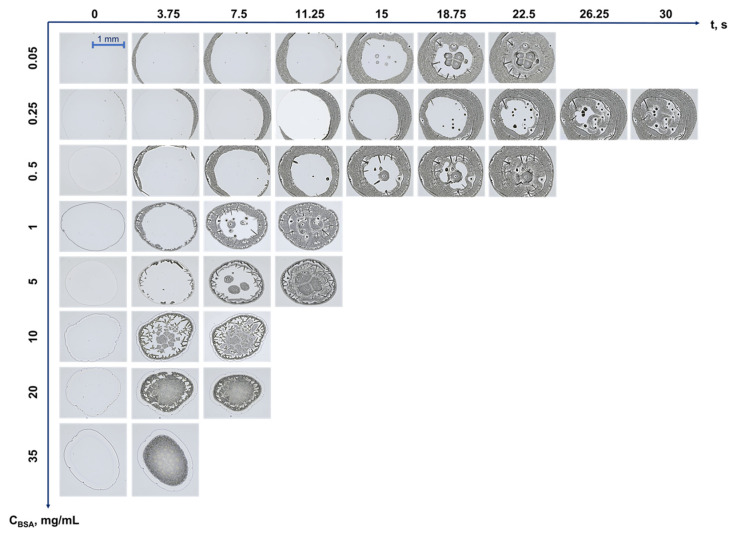
Adsorption structure formation during drying of a drop in a mixture of BSA and NaCl at various BSA concentrations. Time t = 0 s corresponds to the moment of formation of the first crystallization center. C_BSA_ = 0.05, 0.25, 0.5, 1, 5, 10, 20, and 35 mg/mL in 0.9% NaCl. Images were obtained using an optical microscope. All photographs are presented to the same scale. A 1 mm scale bar is shown on the first image.

**Figure 3:**
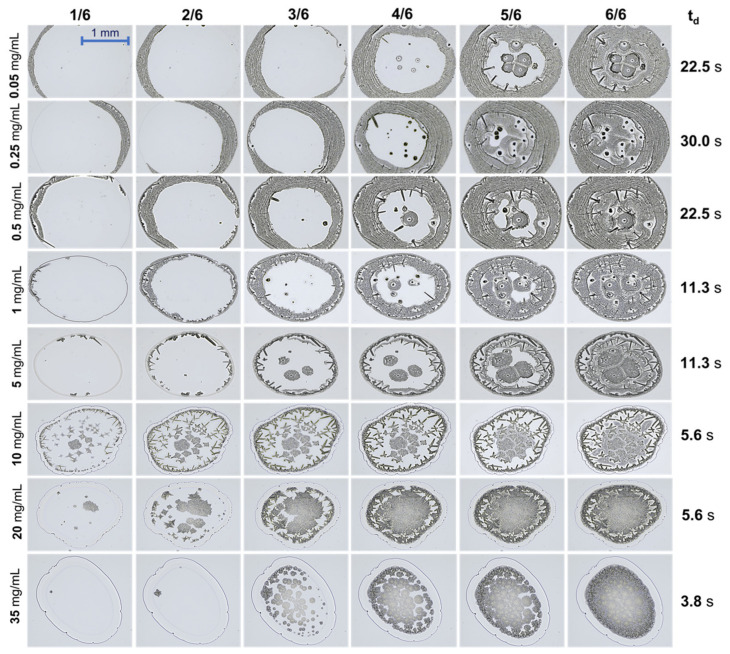
Adsorption structure formation during drying of a BSA–NaCl mixture droplet at BSA concentrations of C_0.05_–C_35_, shown in relative time fractions. Each frame represents 1/6 of the active drying cycle. t_d_(s) indicates the total drying time of the droplet (frame 6/6).

All patterns discussed in this study (Figure 2 and Figure 3) were formed in the colloidal solution during the active stage of droplet evaporation. The active drying cycle began when the first adsorption structures appeared on the glass surface and continued until the drop had completely dried. It accounted for 5–10% of the total drying time. Time t = 0 (the first frame for each concentration in Figure 2) is the start of the active drying cycle. The last frames in each row correspond to the moment when the final pattern of the forming structures was established.

Unique crystal-protein patterns were observed, the appearance of which depended on the concentration of protein, salt, and their ratio to each other as well as on the observation time (Figure 2 and Figure 3). Images illustrating the process of structure formation in a drying drop were obtained based on video recording of the processes in an optical microscope.

**Figure A1:**
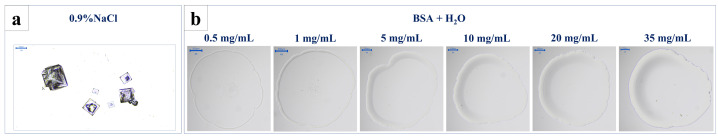
(**a**) Photo of crystals of a 0.9% NaCl solution obtained with an optical microscope. (**b**) Photo of sediments of BSA solutions in distilled water at various concentrations CBSA = 0.5; 1; 5; 10; 20; 35 mg/mL, obtained on an optical microscope. In (**a**) the scale bar is 100 µm. All photographs in (**b**) are shown at the same scale. Scale bar 200 µm.

Analysis of the deposits formed by BSA and NaCl components separately reveals the absence of the complex protein-crystalline patterns characteristic for the combined BSA + NaCl system. In the absence of BSA, only individual NaCl crystals or small clusters are observed. Pure BSA solutions in distilled water form uniform amorphous films without internal structural elements across the entire investigated concentration range of C_BSA_ = 0.5–35 mg/mL (Appendix A, Figure A1).

Figure 2 shows a tendency for all BSA concentrations: as the concentration increased, the active drying cycle of the drop shortened from 30 s for C_0.25_ to 11.25 s for C_1_ and to 3.75 s for C_35_. Thus, an increase in BSA concentration causes accelerated drying of the droplets as a whole. In Figure 2, the concentration of C_0.05_ did not fit into the general tendency. We showed this to demonstrate the concentration threshold nature of the process.

The nature and type of BSA–NaCl structures depended on the initial concentrations of BSA. At low concentrations of C_<0.005_, no protein precipitates formed.

At a concentration of C_0.005_, a transitional state was observed, in which ‘seeds’ of arcs formed by small ordered structures appeared. At concentrations ranging from C_0.05_ to C_1_, the formation of ensembles of outer concentric rings began during the active drying cycle. Then, NaCl crystals formed in the center of the drop, surrounded by concentric inner rings. In parallel, single-ray outgrowths could arise—‘spikes’ 96–230 µm in size, growing from the outer rings to the center of the drop.

When the BSA concentration was increased to C_5_, the outer rings were absent. At the edges of the drop, the rings were replaced by growths of long spikes, reaching ~300 µm, growing from dendritic structures toward the center of the drop. Inner rings were formed with a four-ray polycrystal in the center.

At C_10_, the inner rings were no longer present, and four-ray polycrystals emerged, ranging from 50 to 300 μm in length along the main longitudinal axis. Near the center of the droplet, these four-ray polycrystals gave rise to structures resembling fractal patterns. The largest had a longitudinal axis size of 250–300 μm, with first-order branches reaching ~90 μm and second-order branches reaching ~20 μm.

When the concentration reached C_20_, the fractal-like structure disappeared and multi-ray formations appeared. A rim of BSA dendrites with outgrowths of multi-ray polycrystals of BSA–NaCl compounds 50–70 μm in size was observed at the edges of the droplet. Toward the center of the droplet, the number of directional rays in the crystals increased.

At a BSA concentration of C_35_, the drop consisted of an outer BSA rim with a thickness of 65 μm and an inner part that was uniformly filled with radially symmetric, multi-rayed, dendritic ‘chrysanthemum-like’ structures 45–155 μm in diameter. These ‘flower-like’ patterns are presented below. With increasing BSA concentration in the range of C_10_–C_35_, an increase in the thickness of the protein ring at the droplet edge was observed, reflecting the ‘coffee-ring’ effect.

Figure 3 shows the stepwise formation of patterns at each BSA concentration, starting from the formation of the first crystallization center and ending with the final pattern. While Figure 2 presents the drying events in absolute time (s), Figure 3 represents them as relative time fractions. This approach allows for consistent tracking of the locations of the ‘seeds’ of emerging structures and the growth process of distinct patterns at different concentrations.

Up to BSA concentrations of C_0.05_–C_1_, rings were the dominant pattern. During the first half of the active drying cycle (the first three frames), droplet drying proceeded from the periphery toward the center, with successive formation of the outer rings. We observed the process of sediment ring formation occurring simultaneously in space and time. The outer rings formed non-uniformly: they began either on the left or the right, and after a short period, the ring closed. During this time, the growth of subsequent rings began (Figure 2 and Figure 3; Supplementary Materials, Video S1: Pattern formation in a drying droplet at C = 0.5 mg/mL; Video S2: Formation of outer concentric rings at C = 0.5 mg/mL). The closer to the center, the smaller the ring radius and the shorter the time required for closure. The asymmetry of the pattern is determined not by the adhesion properties of BSA but primarily by the geometry of the contacts between the sample suspension and the glass substrate as well as by the uneven drying along the edges of the droplet.

For BSA concentrations of C_0.05_–C_5_, pattern formation began already in the first 1/6 of the drying cycle (first frame), proceeding from the periphery toward the center and occurring evenly over time. For a droplet at C_10_, the overwhelming majority of ‘seeds’ had already formed in the first frame, followed by a slow process of their evolution leading to the formation of the final pattern. At a concentration of C_35_, the first 1/3 of the cycle (frames 1 and 2) contained the same single ‘seed’, which developed slowly over time. Only from the midpoint of the drying cycle (frame 3) did multiple ‘seeds’ begin to form, accompanied by a rapid drying of the entire droplet surface. This behavior is likely related to changes in the internal state of the BSA–NaCl complexes and the viscosity of the droplet.

Table 1 presents the adsorption structures and the BSA concentrations at which their formation is possible.

### 2.3. Characteristics of Outer and Inner Rings, Spikes, and Salt Crystals

**Figure 4:**
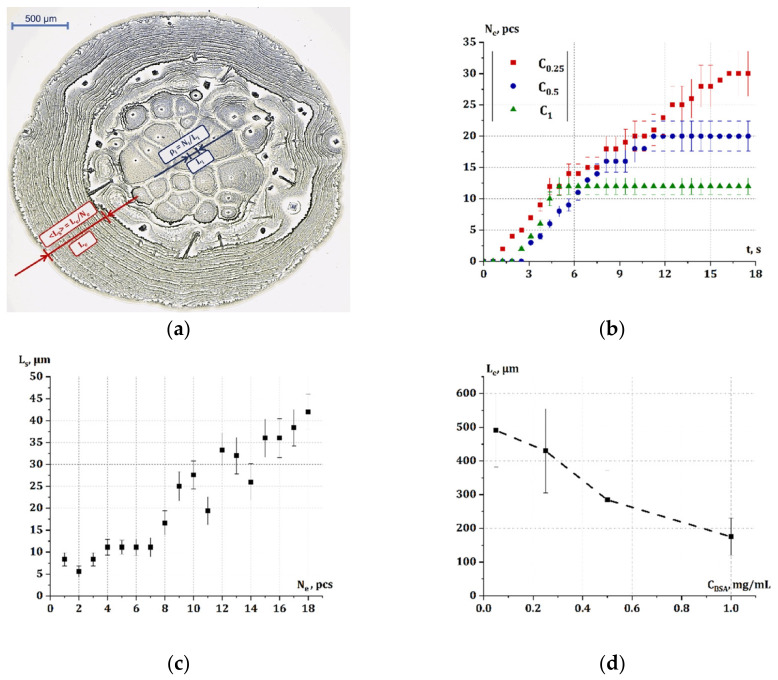
(**a**) Image of the sediment of a dried droplet of a BSA + NaCl solution (C_0.5_). (**b**) Dependence of the number of formed rings of the outer ensemble (N_e_, pcs) on the droplet drying time measured from the moment of appearance of the first crystallization center (t, s) for BSA solution concentrations of C_0.25_, C_0.5_, and C_1_; (**c**) spatial spacing between the initiation points of successive rings (Ls, µm) of the outer ensemble as a function of the ring number (N_e_, pcs); (**d**) dependence of the width of the outer ensemble (L_e_, µm) on the BSA concentration.

In our experiments, the formation of outer rings during droplet drying were recorded in the BSA concentration range of C_0.05_–C_1_. The features of the development of the inner rings were also noted. It was experimentally established that the formation of outer rings proceeds from the droplet periphery toward its center. In contrast, the inner rings expand from the crystallization center toward the droplet edge. Using the obtained images of dried droplets, a quantitative assessment of the characteristic sizes of the outer and inner assemblies of concentric rings was performed. Figure 4a shows an image of a droplet with a concentration of C_0.5_ indicating the average number of rings in the outer (N_e_) and inner (N_i_) assemblies; the average characteristic size of the outer (L_e_) and inner (L_i_) assemblies of concentric rings; the average ratio of the characteristic size of the outer ensemble to the number of rings it contains (<L_s_> = L_e_/N_e_), where (L_s_, µm) is spatial spacing between the initiation points of successive rings of the outer ensemble; and the density of rings in the inner assemblage, defined as the ratio of the number of rings to the characteristic size of the assemblage (ρ_i_).

#### 2.3.1. Outer Rings

The dependences of the parameters of the outer rings are shown in Figure 4b–d.

With an increase in BSA concentration from C_0.25_ to C_1_, the maximum ring quantity decreased from 30 to 12 (Figure 4b). The time step for ring formation was independent of the ring number, which is discussed in the Discussion section. When moving from the droplet edge toward the center, the spatial spacing between the initial points of appearance of successive rings L_s_ increased (Figure 4c). Concurrently, with increasing BSA concentration, the width of the outer ensemble (L_e_) decreased: it was 490 µm at a concentration of C_0.05_ and 175 µm at a concentration of C_1_ (Figure 4d).

#### 2.3.2. Inner Rings and Structures

The inner structures of the droplet consist of various forms of self-organization, and their morphology changes with BSA concentration. At BSA concentrations up to C_5_, the inner area of the droplet was occupied by groups of concentric rings with an individual mono- or four-ray crystal at the center, surrounded by a molecularly depleted zone from which crystals extended to form the inner ring ensembles.

**Figure 5:**
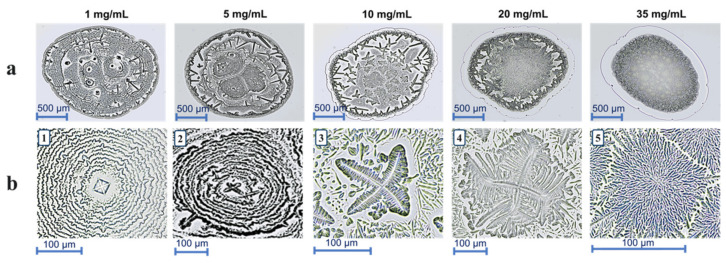
Dynamics of the development of self-organization centers of ordered structures: (**a**) Droplets of BSA solutions at concentrations of C_1_–C_35_. (**b**) Dynamics of intermediate inner structure development: a monocrystal surrounded by concentric (inner) rings (**1**); a four-ray crystal surrounded by concentric rings (**2**); a second-order fractal four-ray polycrystal (**3**); a four-ray polycrystal with second- and third-order fractal-like structures (**4**); radially symmetric branched formations—dendrites extending uniformly from a single center in all directions (**5**).

Figure 5 shows the evolution of the inner self-organization centers of BSA–NaCl structures at different protein concentrations. The top row (Figure 5a) presents optical images of dried droplets at concentrations ranging from C_1_ to C_35_ within a 2.4 mm field of view. With increasing BSA concentration, a qualitative restructuring of the adsorption sediment morphology was observed: from structures with pronounced inner concentric rings (C_1_ and C_5_) to fractal-like structures (C_10_ and C_20_) and finally to radially symmetric dendritic chrysanthemum-like patterns (C_35_). At BSA concentrations above C_10_, the concentric rings disappeared, and the deposit structure was determined by the formation of fractal-like structures, radially symmetric multi-ray polycrystals, and a thickened protein ring at the droplet periphery.

The bottom row (Figure 5b) shows enlarged images of characteristic internal structures at different BSA concentrations. At low concentrations (up to C_1_), a single NaCl monocrystal (1) was observed at the center of concentric ring ensembles. With increasing BSA concentration to C_5_, the monocrystal was replaced by a four-ray polycrystal (2). At C_10_, four-ray polycrystals with fractal-like structures of higher orders (3,4) formed without surrounding rings. At the highest concentration (C_35_), radially symmetric dendritic BSA–NaCl structures were observed, originating from a single point and extending uniformly in all directions (5). In addition, directed single-ray polycrystals (‘spikes’) were observed at concentrations up to C_10_ (Figure 5b). These structures displaced the surrounding rings, forming loop-like ensembles and local depletion zones.

**Figure 6:**
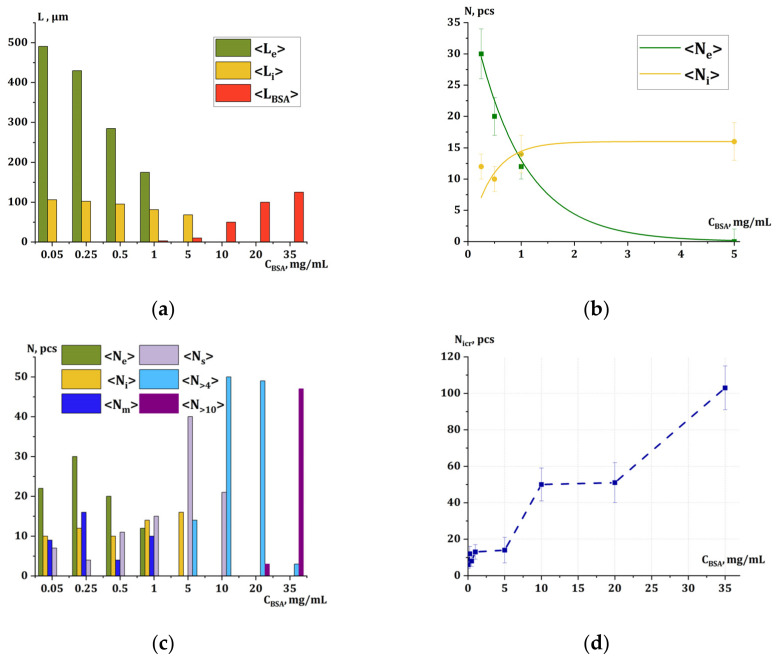
Changes in the typical parameters of absorbed structures as a function of BSA concentration in the range 0.05–35 mg/mL: (**a**) thicknesses of the outer (L_e_, µm) and inner (L_i_, µm) concentric ring ensembles and thickness of the outer transparent protein ring (rim) (L_BSA_, µm); (**b**) number of rings in the outer (N_e_, pcs) and inner (N_i_, pcs) ensembles; (**c**) diagram showing the number of rings in the outer (N_e_, pcs) and inner (N_i_, pcs) ensembles, number of monocrystals (N_m_, pcs), number of single-ray polycrystals (N_s_, pcs), number of four- and multi-ray polycrystals (N_>4_, pcs), and number of multi-ray conglomerates (N_>10_, pcs); (**d**) number of centers of internal crystallization ensembles (N_icr_, pcs) as a function of BSA concentration(C_BSA_, mg/mL).

Inner ring assemblies began to form at the same concentrations as the outer ones and were still observed at C_5_, when the outer rings were already absent. Their number remained nearly unchanged, with only a slight increase (by a factor of 1.2) at initial concentrations up to C_1_. At C_5_, their number remained the same, whereas at C_10_ mg/mL, the inner ring ensembles were no longer formed (Figure 6a,b). When moving from the inner crystal toward the periphery of the inner ensemble, the spacing between the initial points of successive rings increased, similarly to what was observed for the rings of the outer ensemble (Figure 4c).

The width of the outer protein ring increased with increasing BSA concentration. At BSA concentrations of C_1_ and C_5_, the protein ring was virtually invisible, whereas at concentrations greater than C_10_, its width reached 50–150 µm (Figure 6a).

Figure 6c shows a diagram of the number of structures in the topographic pattern of the deposit—the number of rings in the outer and inner ensembles, the number of spikes, and the number of centers of the internal crystallization ensembles. At concentrations where the mass fraction of NaCl molecules exceeded that of BSA molecules (up to C_10_), ring ensembles (outer and inner) were formed in the topographic pattern. However, at a BSA concentration of C_5_, the outer ring ensembles were absent, and the monocrystals were replaced by four-ray polycrystals. The number of spikes oriented from the droplet periphery toward the center sharply increased (2.7-fold), as did their length (Figure 6c).

At comparable mass fractions of NaCl and BSA molecules (C_10_ (BSA fraction 1% (C_20_), NaCl 0.9%)), the formation of ring ensembles ceased. Spikes remained only at the droplet periphery, with their number reduced by half, while the entire inner area was occupied by four-ray polycrystals. Their number increased 3.6-fold compared to that at a concentration of C_5_ (Figure 6c,d).

At high concentrations (C_20_ and C_35_), where the mass fraction of BSA molecules exceeds that of NaCl, an increase in the width of the protein ring was observed (Figure 6a). Single- and four-ray polycrystals transformed into conglomerates with defined growth directions at C_20_. At C_35_, the sediment pattern was almost entirely composed of radially symmetric, branched dendrites (Figure 6c).

The stepwise increase observed in the curve in Figure 6d indicates a staged redistribution of BSA. Ring ensembles are replaced by spikes or four-ray polycrystals in the C_5_–C_10_ range, followed by branched dendritic patterns at C_20_–C_35_.

### 2.4. Adsorption Structures at the Millisecond and Micrometer Scales: Analysis Using Electron Microscopy and Quantitative Topographic Analysis

Scanning electron microscopy was used to perform a topographic analysis aimed at investigating the surface relief of structures in the sediment as well as a quantitative analysis of the composition of the deposits in dried droplets.

#### 2.4.1. Outer Rings ($C_{BSA}=\left(0.25-1\right)\frac{mg}{mL}$)

**Figure 7:**
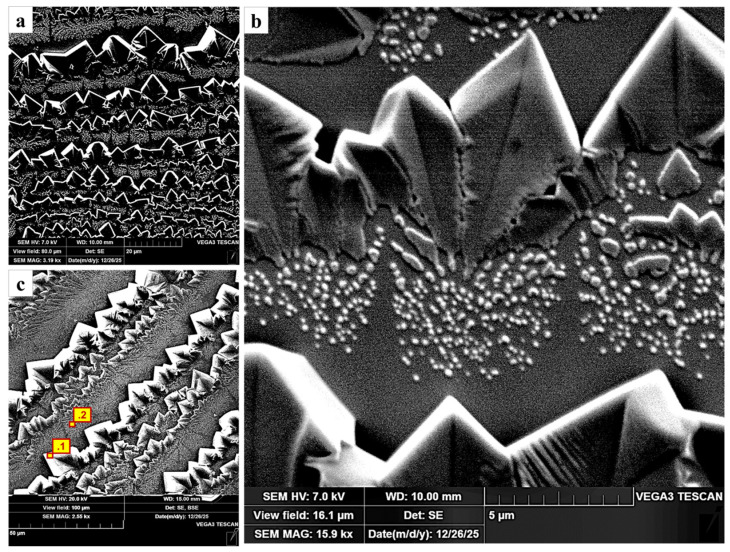
SEM images of adsorption ordered structures. Bilayer nature of the outer ring ensembles at C_0.5_, obtained by scanning electron microscopy: (**a**) field of view 80 µm; (**b**) field of view 16.1 µm; (**c**) field of view 100 µm. Yellow markers ‘.1, .2’ indicate the locations of points of material analysis by SEM/EDS.

Figure 7 shows ensembles of outer rings whose crystal growth was directed from the periphery toward the center. As the distance from the droplet edge increased, the spacing between adjacent rings also increased (Figure 7a). The spatial step between the onset points of formation of successive rings in the outer ensemble (L_s_, µm) as a function of the ring number (N_e_, pcs) was previously shown in Figure 4c. The observed increase in distance is associated with both an increase in ring width and an increase in the spacing between adjacent rings. This effect is not resolved in optical images due to the limited visibility of BSA conglomerate sectors and is therefore only detected in scanning electron microscopy (SEM) images (Figure 7).

During ring formation, the first stage involved the aggregation of BSA particles that adsorbed onto the surface as balls structures (Figure 7, Supplementary Materials, Video S1: Pattern Formation During Droplet Drying at C_0.5_ and Video S2: Formation of Outer Concentric Rings at C_0.5_). The average size of a single aggregate ball-like particle was 230 ± 30 nm (Figure 7b). These aggregates formed self-organized sectors.

The sectors shown in the SEM images are below the salt crystals (Figure 7a–c). This means that sectors with spherical structures arise earlier than crystals. They exhibited different sizes: the area of these sectors varied over a wide range, from ~4 µm^2^ near the droplet edge to ~100 µm^2^ in rings located farther from the edge. Similarly, the number of particles within a sector varied from ~40 to ~1000.

The average density of colloidal particles within the sectors remained nearly constant, amounting to 8.5 ± 1.2 particles/µm^2^, irrespective of the ring’s position. The average spacing between individual sectors was 1.20 ± 0.17 µm, that is, the size remained the same. In the upper part, near the transition to the crystal growth point, the fusion of colloidal particles into groups of 2–4 or more was observed (Figure 7b).

In the second stage, these sectors became the growth points for NaCl crystals (either single or multiple), forming druses oriented toward the direction of droplet drying (Figure 7). It can be assumed that the growth of crystal druses near the BSA aggregated sectors commenced as soon as the local concentration of the NaCl reached a critical value for salt crystallization. The number of crystals within the druses and their sizes can vary and depend on the amount of Na^+^ and Cl^−^ ions available within the advancing front zone.

The final stage of ring formation resulted in bilayer outer rings, featuring an outer layer of aggregated BSA particles and an inner layer of NaCl crystals extending toward the center of the droplet.

Upon moving from the droplet edge toward the center, the crystal lengths increased–by a factor of 5 for rings 1 and 8 (C_0.5_, Figure 7). The spatial step (period) also increased (Figure 4d and Figure 7). However, when comparing the ratios of the spatial step to the width of the crystalline druses for the rings of the outer ensemble, these values were found to be comparable, averaging 2.1 ± 0.4.

**Figure 8:**
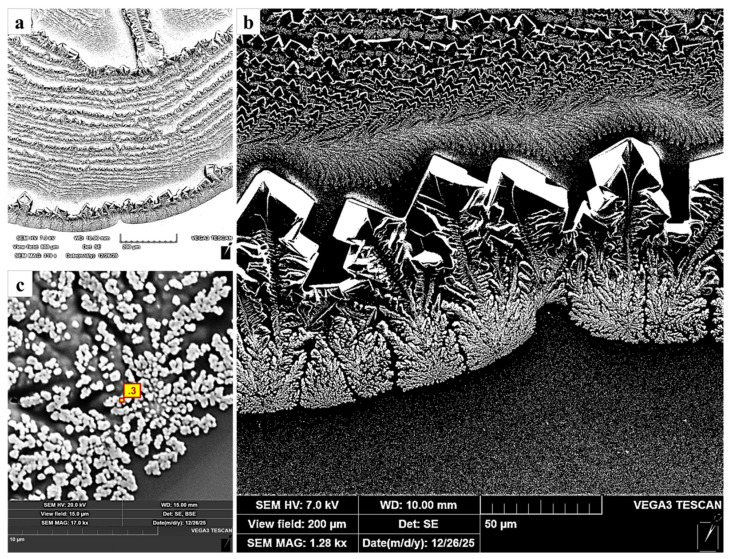
SEM images of dendritic structures at the edge of a droplet (C_0.5 BSA_ = 0.5 mg/mL): (**a**) field of view 800 µm; (**b**) field of view 200 µm; (**c**) field of view 15 µm. The yellow marker ‘.3’ indicates the location of point of material analysis by SEM/EDS.

At C_0.5_ ring formation was observed across the entire droplet. However, depending on the location, the growth base of the crystalline druses varied. At the droplet edge, where the BSA concentration was higher, the growth points of the crystalline druses were not point-like deposits of colloidal particles, as observed during the formation of the outer ring (Figure 7b), but dendritic structures forming a ring around the NaCl crystals (Figure 8). The thickness of such rings in the droplets ranged from 28 to 34 µm; in particular, in Figure 8b, it measured 32 µm. The branches of the dendritic formations were densified toward the exterior and branched toward the crystals.

#### 2.4.2. Inner Rings ($C_{BSA}=\left(0.25-1\right)\frac{mg}{mL}$)

**Figure 9:**
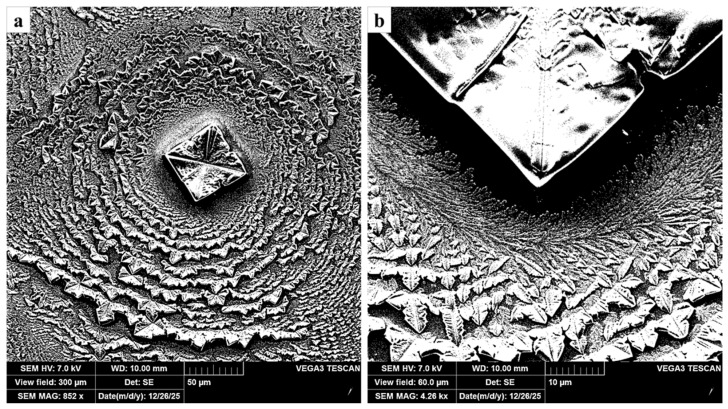
SEM images of the adsorption structures of the inner ring ensembles with a single crystal at the center (C_0.5_): (**a**) field of view 300 µm; (**b**) field of view 60 µm.

Around the single crystal at the center of the inner ring ensemble, distinctive local conditions developed, giving rise to a depletion zone approximately 6–7 µm in width. Adjacent to this zone, dendritic formations appeared, following a curved trajectory that wrapped around the single crystal and also measured 6–7 µm in width. Beyond these densely packed dendrites, a region of oriented polycrystalline lines was observed, which appeared to grow out of one another and were curved away from the central single crystal (Figure 9b).

Only then did the rings of the inner ensemble appear, their formation following the same pattern as in the outer ensembles. The growth of the crystalline druses was always directed toward the droplet drying front: for the outer ring ensembles, from the droplet periphery toward the center (Figure 7a), and for the inner ring ensembles, from the centers of the internal crystallization ensembles outward (Figure 9).

#### 2.4.3. ‘Chrysanthemum-like’ Structures ($C_{BSA}=\left(20-35\right)\frac{mg}{mL}$)

**Figure 10:**
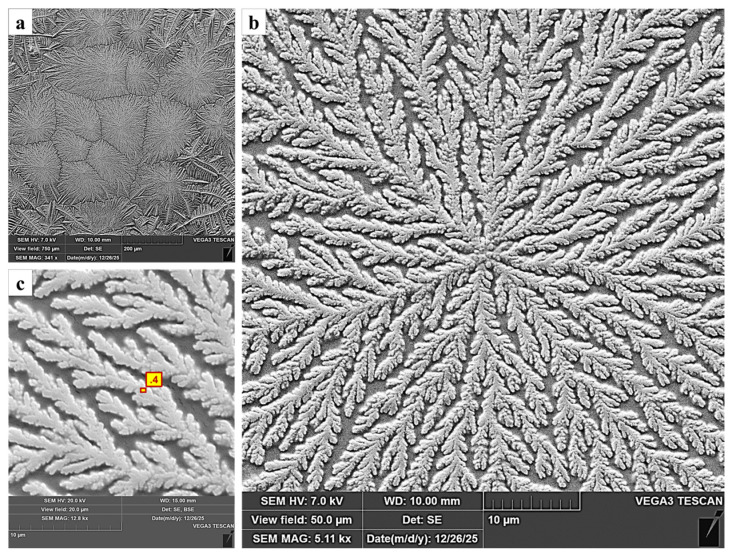
SEM images of radially symmetric, multi-branched dendritic structures (C_35_): (**a**) field of view 750 µm; (**b**) field of view 50 µm; (**c**) field of view 20 µm. The yellow marker ‘.4’ in (**c**) indicates the location of point of material analysis by SEM/EDS.

At a BSA concentration of C_35_, a fundamentally different type of precipitate formed, consisting of dense, branched, radially symmetric multi-ray dendrites (Figure 10) composed of tightly packed elements radiating uniformly from a single center (Figure 10b,c). Their growth occurred continuously in all directions, without a single seed at the center (Figure 10b). A similar type of precipitate began to form at a concentration of C_20_ within the droplet; however, a larger portion of the droplet was occupied by four- or more-ray fractal structures. At C_35_ mg/mL, multi-ray dendrites occupied nearly the entire droplet area (Figure 10a).

At high BSA concentrations, optimal conditions for BSA aggregation are created. The number of BSA molecules increases to such an extent that virtually all NaCl molecules participate in their aggregation; consequently, the resulting BSA aggregates effectively displace the salt molecules. BSA molecules can adsorb onto the growing facets of salt crystals, causing the salt to grow in directions with lower BSA concentrations, which ultimately leads to branching.

**Figure 11:**
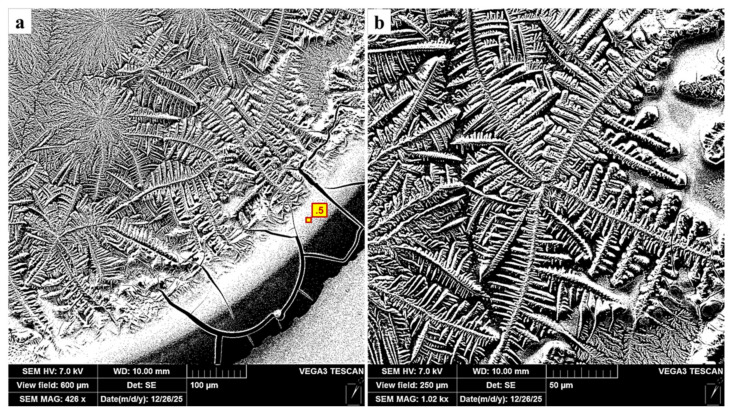
SEM images of BSA + NaCl adsorption structures formed at the drop edge (C_35_): (**a**) protein ring, field of view 600 µm; (**b**) multi-branched fractal-like structure, field of view 250 µm. The yellow marker ‘.5’ in (**a**) indicates the location of point of material analysis by SEM/EDS.

As shown earlier, a protein ring formed at the droplet edge, presented in Figure 11. Between this protein ring and the droplet interior—filled with branched patterns—multi-ray fractal-like structures could form (Figure 11b), resembling palm fronds. Fractal structures were observed at concentrations as low as 10 mg/mL, and at 20 mg/mL, they became the predominant pattern.

At C_10_, the fractal-like structures exhibited four primary rays, with an angle of 90° between successive structures of the 2nd to 4th orders (Figure 5). Fractal patterns have also been observed in the work of other authors [5,10,17,18,19]. As the concentration increases, additional growth directions emerge from the initially diverging rays, each of which retains its fractal character. With a further increase in concentration, these structures evolve into branched patterns (Figure 10).

### 2.5. Elemental Composition Analysis of Sample Structures Using Electron Microscopy

The elemental composition of the sample structures was analyzed at several local points (Table 2): Control point (pure crystal)—a crystal from a NaCl solution without BSA; point 1—the outer corner of a NaCl crystal within the outer ring (Figure 7c); point 2—an aggregate of BSA particles located in a precipitate sector (Figure 7c); point 3—a branched formation at the rim of BSA dendrites (Figure 8c); point 4—a fragment of a branched structure (Figure 10c); and point 5—the protein ring at the drop edge (Figure 11a).

During SEM scanning, the elemental composition was analyzed at each of the indicated points. Table 2 shows the weight and atomic percentages of carbon (C) and nitrogen (N) (characteristic of BSA molecules), sodium (Na) and chlorine (Cl) (characteristic of the physiological saline), silicon (Si) (characteristic of the glass substrate), and oxygen (O). The total contribution of other elements did not exceed 10% of the overall elemental composition at any of the points analyzed.

To estimate the proportion of organic and inorganic constituents in the deposit of dried droplets at a given point, the weight and atomic coefficients of elemental composition, G_W_ and G_A_, were introduced. $G_{W}$ is the weight ratio of the sum of organic components to the sum of inorganic components, $G_{W}=\frac{N+C}{Na+Cl}.G_{A}$ is the atomic ratio of the sum of organic components to the sum of inorganic components, $G_{A}=\frac{N+C}{Na+Cl}.$ The ratios G_W_ and G_A_, or their reciprocal values 1/G_W_ and 1/G_A_, indicate whether BSA or salt predominates at a given point of the adsorption structure.

Analysis of the elemental spectrum of a pure NaCl single crystal (p.c.), obtained by drying a 0.9% NaCl solution on a glass substrate, revealed the absence of carbon (C) and nitrogen (N), with G_W_ = 0 and G_A_ = 0.

The presence of atoms *C* and *N* in the adsorption structures confirms the presence of BSA molecules at the scanned points (points 1–5). The weight and atomic content results showed that both BSA and NaCl were present in all observed ordered structures. However, the ratio of these components varied significantly across the observation points.

At *point 1* (C_0.5_), NaCl predominated by weight on the crystal facet, with the 1/G_W_ ratio being 17 times higher than that at *point 2* on the sector for the adjacent spherical aggregates, where BSA content was predominant.

At *point 5* (C_35_), which corresponds to the protein ring, the combined weight percentage of carbon and nitrogen amounted to 73%, while their combined atomic percentage reached 82%, confirming the predominance of protein in this structure.

Elemental analysis revealed that the aggregates in the ring structures (*point 2*, C_0.5_), at the rim of BSA dendrites (*point 3*, C_0.5_) and in the chrysanthemum-like dendrites (*point 4*, C_35_) had approximately the same quantitative composition, as evidenced by the ratios G_W_ = 4.3 ± 0.4 for weight content and G_A_ = 8.5 ± 2.0 for atomic content, respectively. Furthermore, the chrysanthemum-like dendrites were composed of spherical aggregates similar to those in the outer ring structures.

Thus, the study of the elemental composition using SEM made it possible to reveal the synergism of BSA and NaCl in the formation of a precipitate of two phases (aggregates and crystals) in a direct biophysical experiment. The SEM and EDS measurements constitute a valuable part of the work because they support the coexistence of protein-rich and salt-rich regions and suggest a sequential relationship between BSA aggregation and NaCl crystallization.

## 3. Discussion

In our experimental study we investigated morphology and typical structures of drying-induced BSA–NaCl self-organization.

### 3.1. Droplet Drying of BSA + NaCl + H_2_O: Cycles and Phases

The complete drying cycle of the droplet spans from its initial deposition onto the substrate to the end of the drying process, i.e., until the sedimentary patterns are fully formed on the substrate.

In the phenomenological analysis of the appearance of structures during the drying of a drop, we distinguish two periods: passive and active cycles. *The passive cycle* covers the period from droplet deposition to the appearance of the first adsorbed structures. This cycle lasts for about 95% of the total drying time. During this stage, droplet evaporation occurs, the volume decreases, and the droplet dome subsides. The droplet contact line remains pinned, and the droplet diameter does not change throughout this cycle. This is evidenced by experimental results (Figure 2 and Figure 3 and Supplementary Materials, Video S1: Pattern Formation During Droplet Drying at C_0.5_). During this cycle, the concentrations of both BSA and NaCl in the droplet increase due to the volume reduction caused by evaporation. *The active cycle*—the droplet continues to evaporate, and its volume decreases and at the same time BSA–NaCl structures adsorb onto the substrate. The droplet contact line moves toward the center, and the droplet diameter decreases. By the time the drop dries, all structures will be formed. Due to more intense evaporation at the edges, the solution concentration there reaches a critical point (supersaturation) faster than at the center. This triggers the precipitation of the first microparticles, which then serve as centers for further growth.

The assertion regarding the shift of the droplet contact line is supported by the experimental observation that the outer rings form at the droplet edge and emerge sequentially from the periphery toward the center (Figure 2 and Figure 3 and Supplementary Materials, Video S2: Formation of Outer Concentric Rings at C_0.5_). The droplet edge (contact line) thus progressively moves toward the center from one ring to the next.

The active cycle is divided into two phases.

*Phase 1* of the active cycle: The droplet retains its dome-shaped form and evaporates as a single, intact droplet. Phase 1 begins with the appearance of the first adsorption structures (Figure 2 and Figure 3).

*Phase 2* of the active cycle: The fragmentary drying phase is characterized by planar drying. The droplet transforms into a thin film that dries non-uniformly across its surface, forming small local fragments. Its boundaries separate, giving rise to several distinct small droplets and ‘islands’.

Often, several inner rings form simultaneously (Supplementary Materials, Video S3: “Formation of Inner Concentric Rings at C_0.5_”). This indicates that multiple separate small droplets are drying concurrently in different locations. Thus, drying during the second phase does not proceed directionally toward the center but rather occurs fragmentarily and simultaneously in multiple sites.

**Figure 12:**
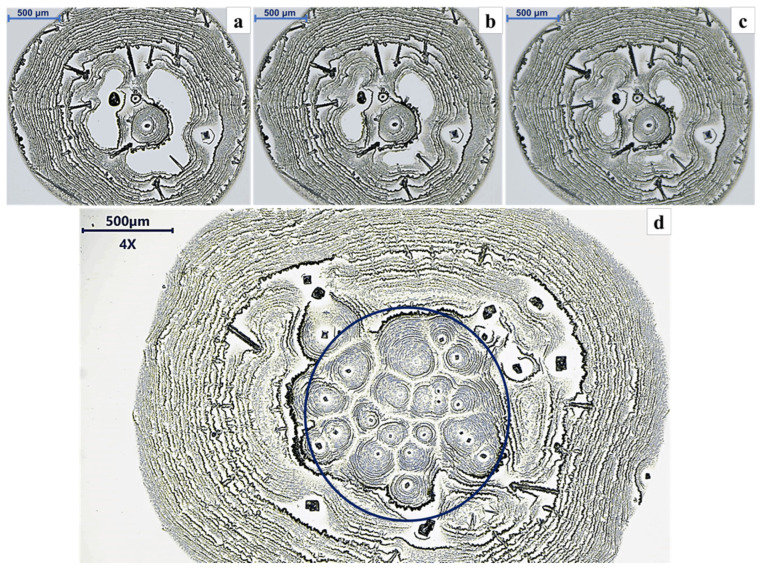
Drying process of a droplet (C_0.5_) and the formation of complex adsorption structures: (**a**) outer rings continue to form, the number of spikes has increased, loops begin to form around them, and inner rings have already appeared; the droplet has split into two fragments, initiating fragmentary drying; (**b**) both undried droplet fragments have shrunk and now resemble curved pear-like shapes; (**c**) the boundaries of the droplet fragments have closed, and they have split into three components; (**d**) both phases of the active cycle of BSA–NaCl structure formation: the first phase—beyond the blue ring; the second phase—inside the blue ring.

Results of the second phase is conditionally shown in Figure 12d inside the blue ring (Supplementary Materials, Video S3: Formation of Inner Concentric Rings at C_0.5_).

While clear boundaries exist between the passive and active cycles, the phases of the active cycle do not have such distinct demarcations. They can overlap and intertwine, giving rise to similar structures in either phase. For example, in Figure 4b, an inner ring has already formed, while loops are still in the process of forming.

Table 3 shows the temporal relationships between the stages and phases of droplet drying for different BSA concentrations.

### 3.2. Qualitative Interpretation of the Emergence of Ordered Structures

#### 3.2.1. Formation of Outer Rings

In our experiments, we observed an interesting and original effect: the formation of ensembles of ordered periodic rings in the BSA–salt system at the edge of a drying droplets (Figure 3, Figure 4a, Figure 7 and Figure 12, Supplementary Materials, Video S2: Formation of Outer Concentric Rings at C_0.5_), $C_{BSA}=\left(0.005-1\right)\frac{mg}{mL}$. It should be noted that single rings (rims) are usually studied in the BSA–salt system (like coffee rings) [3,8,10,12,23].

**Figure 13:**
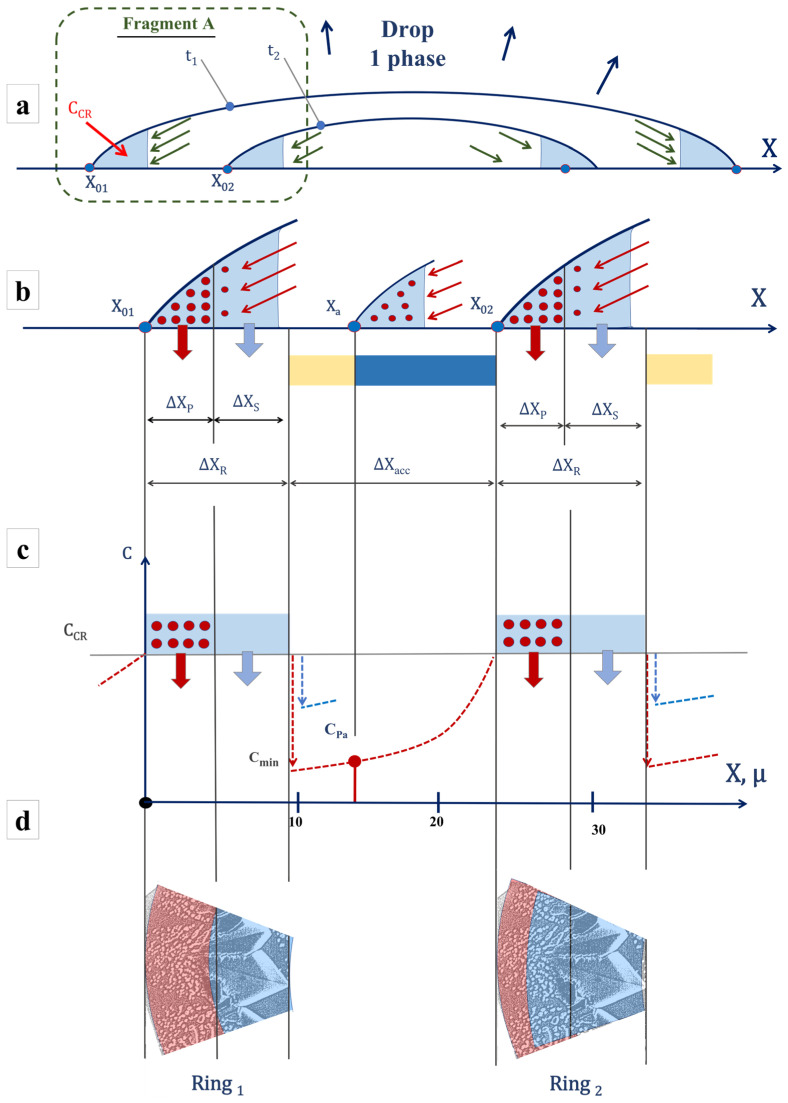
Conceptual schemes of outer rings during the first phase of the active cycle. (**a**) Schematic representation of a droplet during the first drying phase at two time points, t_1_ and t_2_; point X_0_ shifts as evaporation progresses. (**b**) Enlarged fragment corresponding to region A in (**a**). (**c**) Schematic variation in the concentrations of BSA and NaCl near the droplet edge. (**d**) Formation of two rings on the substrate; blue indicates NaCl; red indicates BSA. Red arrows indicate the location of the BSA structure. Blue arrows indicate the location of the salt structures.

We propose a phenomenological approach for the qualitative interpretation of the formation of periodic ring structures (Figure 13). The conceptual schemes is described in Figure 13. Points X_01_ and X_02_ (Figure 13) indicate the positions of the contact line at the moments of precipitate deposition and ring formation (t_1_ and t_2_, respectively). ΔX_P_ and ΔX_S_ denote the regions where BSA aggregates precipitate and salts crystallize, respectively. ΔX_R_ represents the ring formation zone and its width.

In Figure 13b schematically shows a magnified view of the droplet region in the immediate vicinity of point X_01_ (4–10 μm) at the droplet corner. The concentration of BSA particle aggregates (shown in red) at the corner is increased due to capillary flows of particles (arrows). Moving toward the center, BSA concentration decreases, although they remain present. Point X_a_ is an intermediate location to which the droplet contact line (the droplet corner) has advanced by time t_a_. At this moment, the concentrations of BSA and NaCl have not yet reached their critical values (C_CR_). Their accumulation continues via capillary flows and droplet evaporation (Figure 13c). C_Pa_ represents the accumulated concentration of BSA at the droplet corner by the time t_a_, when the contact line has shifted to point X_a_. The area between the precipitate site and point X_a_ (at time t_a_) represents the dried substrate and is shown in yellow. The area beyond point X_a_ (at time t_a_) corresponds to the wet region of the droplet and is shown in blue. As the contact line (the droplet corner) advances, the yellow sector extends to the right, while the blue sector correspondingly shortens.

As the droplet continues to dry, its contact line advances from point X_a_. Upon reaching point X_02_, where the concentrations of BSA particles and salts have risen to their critical values (C_CR_) (Figure 13c), the next precipitate deposition occurs, forming an adjacent ring (Figure 13d). The red dashed line represents the change in BSA particle concentration at the successive positions of the advancing droplet corner (the contact line). The resulting rings consist of two layers: an outer layer composed primarily of BSA (shown in light red) and an inner layer composed primarily of salt crystals (shown in blue).

It is assumed that the processes described below occur at small scales: distances of 3–40 μm and time intervals of 15–500 ms. In the droplet corner, within a small vicinity of point X_01_ (4–10 μm), the following processes unfold.

Due to droplet evaporation and capillary flows, the concentrations of BSA particles and salts increase sharply, causing their local concentrations at the droplet corner to exceed the critical C_CR_ values. High concentrations of BSA and salts lead to interactions between the two components. NaCl molecules promote the aggregation of BSA molecules by altering their local charge [37,38,39,40].

BSA particles can combine into large aggregates. While the initial molecular dimensions were on the order of 6–15 nm [13], after aggregation their sizes reach 200–240 nm. BSA aggregates, in turn, act as catalysts for salt nucleation [5,41]. As a result, both BSA particle aggregates and salts precipitate onto the substrate (thick red and blue arrows, Figure 13b,c), which is well-illustrated in the SEM images (Figure 7).

In the conceptual model (Figure 13b), the BSA aggregates and NaCl crystals within the ring are separated: BSA particles are located closer to the corner, while the salt crystals are shifted toward the center of the droplet. This corresponds to the Figure 7. This is caused by a sharp local increase in BSA concentration, which induces phase separation; the massive BSA aggregates displace the NaCl molecules, resulting in their spatial segregation [5,10,13]. Such separation leads to the formation of bilayer rings (Figure 7 and Figure 13b).

**Figure 14:**
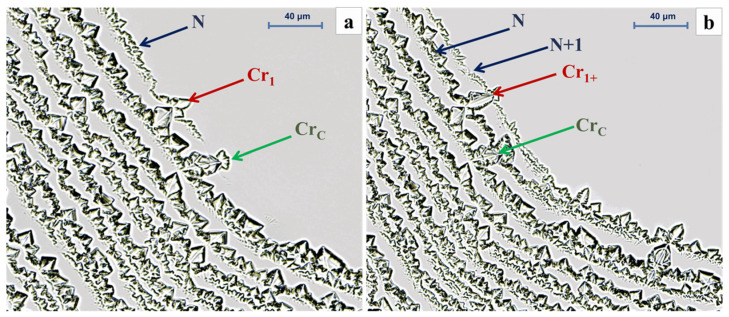
NaCl crystallization process during the formation of outer rings in Phase 1 of the active cycle. (**a**) The moment of appearance of the N ring, (**b**) the moment of appearance of the N + 1 ring. N—the initial ring under consideration; N + 1—the next formed ring; Cr_1_—initial morphology of the crystal of interest (red arrow); Cr_1+_—final morphology of the same crystal; Cr_C_—control crystal for comparison (green arrow).

BSA precipitates first, followed by the salts. For example, in Figure 14, rings N and N + 1 begin with a zone of BSA aggregate precipitation. These aggregates serve as nucleation sites for salt crystals, which then continue to grow inward into the solution. The average thickness of the double ring is approximately 10–20 μm (Figure 7c). Detailed parameters of the rings and trends in their variation depending on the initial BSA concentration are described above.

After ring formation, a zone of gradual accumulation of BSA aggregates and salts at the droplet corner follows $∆X_{acc}=2-20\;\mu m$. It corresponds to the distance between adjacent rings (Figure 2, Figure 3 and Figure 7). Figure 4c shows that the distance L_s_ from the beginning of one ring to the beginning of the next increases with ring number. It should be noted that L_s_ increases due to both the increase in ring width ($∆X_{R}=∆X_{P}+∆X_{S})$ and the increase in the inter-ring distance $∆X_{ACC}$. This effect is subtle in optical images because the sectors of BSA conglomerates are not clearly visible. However, it was distinctly observed in SEM images (Figure 7).

The contact line shifts closer to the droplet center, and in this region, due to continued droplet evaporation and capillary flows, the concentrations of both components begin to increase at the droplet corner. In the BSA + NaCl system, ring formation generally consists of BSA precipitation and subsequent growth of salt crystals. BSA precipitation is a short process, while subsequent crystal growth takes 500–600 ms.

After the precipitation of BSA particle aggregates and NaCl, their concentration at the droplet corner decreases to a certain baseline value, C_min_ (Figure 13c).

When the BSA and NaCl concentrations increase to critical values (red dashed line, Figure 13c), the contact line shifts to a point X_02_, and the processes described above repeat. Thus, the phenomenon of discrete precipitation of rings arises. The period of one ring formation cycle is ΔT_cycle_ ≈ 600–800 ms. The ratio between the cycle period and the ring formation time can vary widely, depending on the component concentrations and droplet drying conditions. Thus, the phenomenon of discrete ring formation occurs.

It has been experimentally established that salt crystal growth can continue after precipitation (Figure 14). The process of salt crystal growth shown in Figure 14 can occur in the ΔX_acc_ region. Crystal Cr1 formed on ring N and continued to grow in time and space until the emergence of the next ring, N + 1. The growth duration of this particular crystal, Cr1, was 410 ms. That is, the crystal grew as the droplet contact line shifted and ceased growth upon the appearance of the next ring.

However, many crystals cease growth earlier, before the next ring appears. These growth durations can be on the order of 100–200 ms or similar timescales. Figure 8 shows crystals that grew differently: some over short periods and others continuing until the formation of subsequent rings.

Figure 14 clearly illustrates the double-layer structure of the rings. In Figure 14a, ring N begins to emerge with a thin belt of BSA particle aggregates. In Figure 14b, ring N has already become bilayer—a thin BSA belt and a layer of NaCl crystals—while the next ring, N + 1, again starts with a thin belt of BSA particles.

#### 3.2.2. Formation of Inner Rings

Inner rings have a different shape and size compared to the outer ones. They appeared primarily during the second phase of the active cycle and completed the formation of sedimentary structures in the drying droplet. The formation of inner rings took approximately 25–30% of the active cycle time. By this stage, the droplet had become thin and dried not as a single entity, but fragmentarily, in small islands.

**Figure 15:**
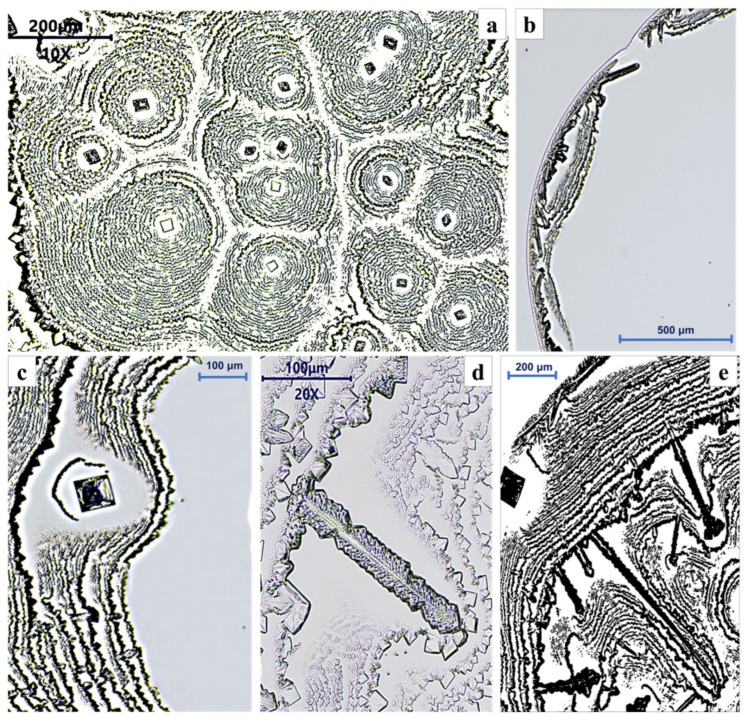
Formation of loops and internal rings, NaCl crystal seeds: (**a**) several internal rings. (**b**) the beginning of the active cycle: after the emergence of a NaCl crystal seed, an ensemble of loops forms around it; (**c**) loops of various configurations around an individual crystal; (**d**,**e**) loops around spikes of different sizes. Loops always form around the depletion zone, followed by the outer rings.

Large NaCl crystals, which formed at the centers of drying droplet fragments, played a decisive role in the formation of inner rings. These crystals marked the onset of Phase 2 of the active cycle. A salt-depleted zone emerged around them (light area in Figure 15a). As the crystals grew, a region with a reduced concentration of the main substance (the depletion zone) developed around the growing facet.

The mini-droplet zone began to dry outward from the already formed crystals. This resulted in the emergence of several contact lines radiating from the central salt single crystals. The boundary between the depletion zone and the bulk zone was formed.

The first inner ring formed around the depletion zone surrounding the crystal, and then, the ensemble of these rings grew from the center to the periphery [5]. The entire cascade of inner rings grew from the crystal, repeating the bizarre geometry of the contact line of the drying mini-droplet. Detailed parameters of the inner rings are described in the Results Section 2.3.2.

### 3.3. The Formation of Ordered Structures in the Sediment as a Result of Synergistic Processes

**Figure 16:**
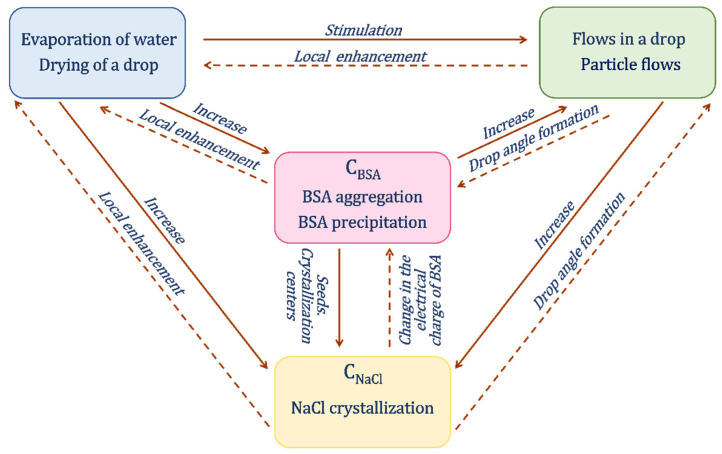
Synergy of processes during the self-organization of sediment in a drying droplet.

The ordered patterns illustrating self-organization in adsorbed precipitates (Figure 5, Figure 7, Figure 9, Figure 10, Figure 11 and Figure 15) arise due to the synergy of heterogeneous processes in the BSA + NaCl + H_2_O system (Figure 16). In studies modeling processes in an evaporating droplet, emphasis is placed on the evaporation of free water and the relative contributions of capillary, diffusive, and convective flows [18,25,26] as well as on aggregation/crystallization [8,14,20,23,29].

Molecular interactions between BSA and NaCl play a crucial role in precipitate formation. Specifically, the coordination of local spatiotemporal scales of the processes involving these molecules is key to the formation of ordered precipitate patterns (e.g., the formation dozens of rings, ‘chrysanthemum-like’ structures, etc.).

A necessary condition providing the physical basis for BSA and NaCl aggregation is the reduction of repulsive forces between negatively charged BSA molecules due to their shielding by Na^+^ ions. Upon a critical increase in salt concentration (supersaturation) during water evaporation, each BSA molecule is subjected to an ion assault, resulting in multiple-collision interactions that lead to protein aggregation. Concurrently, ions bound to the protein serve as ‘anchor points’ for subsequent crystal formation.

As a result, the synergistic interaction of BSA, Na^+^ ions, and Cl^−^ ions enables the formation of ordered patterns in the precipitates of the drying droplet.

## 4. Materials and Methods

All stages of the experiment are presented in Figure 1.

### 4.1. Objects of Study and Sample Preparation

Bovine serum albumin (BSA, Fraction V, BioClot GmbH, Bavaria, Germany) was used as the protein component. A stock solution with a concentration of 35 mg/mL (526.31 μM) BSA (C35) was prepared by dissolving 350 mg of dry BSA in 10 mL of physiological saline (0.9% NaCl). For complete dissolution of the protein, the solutions were kept for 18–24 h at 4 °C. After complete dissolution of BSA and NaCl, working BSA + NaCl solutions with concentrations C_i_ (i = 0.05, 0.25, 0.5, 1, 5, 10, 20, and 35 mg/mL), corresponding to 0.75, 3.76, 7.52, 15.04, 75.19, 150.38, and 300.75 μM, respectively, were prepared by serial dilution of the stock solution C35 with physiological saline. Physiological saline (0.9% NaCl; Grotesk LLC, Saint Petersburg, Russia) was used as the solvent for all dilutions. The NaCl concentration in all solutions was 9 mg/mL (154 mM). The prepared solutions were used within several hours of preparation.

After thorough mixing, a 1 μL droplet of the BSA + NaCl working solution was deposited onto glass slides (Micromed-SPb, Saint Petersburg, Russia) using a micropipette. Then, 1 μL droplets of the working BSA + NaCl solutions were deposited onto glass slides (Micromed-spb, Russia) using a micropipette. The slides were rinsed with distilled water and ethanol prior to use. Droplet drying was carried out in a horizontal position at room temperature (22 °C, 295 K) and at a relative air humidity of 65 ± 70% under ambient laboratory conditions. Dry samples were examined after 15–24 h, as is customary when working with dried droplets [42].

### 4.2. Optical Microscopy (Images and Video)

BSA + NaCl droplet samples were examined using a laboratory direct optical microscope (ADF U300B, NINGBO SUNNY INSTRUMENTS Co., Ltd., Yuyao, China) equipped with a digital microscopy camera (ADF ULTRA 09, ADF OPTICS Co., Ltd., Yuyao, China) connected to a computer. Photographs and video recordings of pattern formation in drying droplets were acquired at objective magnifications of 40×–400× with a frame rate of 30 frames per second. The characteristic dimensions of the evolving structures and the morphological parameters of the precipitates were evaluated using ADF Image Capture software (OPTICS Co., Ltd., Yuyao, China, version x64, 4.11.23385.20230918) and ImageJ (National Institute of Health, Bethesda, MD, USA, version 1.54t).

### 4.3. Scanning Electron Microscopy

A Tescan VEGA3 LMU scanning electron microscope (SEM) (Tescan, Brno, Czech Republic) equipped with an energy-dispersive X-ray microanalysis (EDS) system was used to study the micrometer-scale topography and elemental composition of sedimentary structures. After complete drying of 1 μL droplets of solutions at various concentrations, the samples were sputter-coated with a thin gold layer to prevent charging during SEM imaging. Scanning was performed in various regions of the dried droplets for concentrations C_0.5_, C_1_, C_5_, C_10_, and C_35_. Precipitate morphology was analyzed from the obtained SEM images, and the elemental composition of characteristic structures was determined using EDS spectroscopy. Spectra were recorded at selected points on the surface corresponding to specific morphological features. The analysis determined the relative contents of the main elements in the system: C and N, characteristic of protein molecules; Na and Cl, associated with the crystalline NaCl phase; as well as O and other elements present in the molecules and the substrate, each with a content below 10%. Comparison of SEM images with EDS elemental maps allowed us to establish a correlation between the morphology of the structures and their chemical composition.

### 4.4. Statistical Analysis

The sample size for each concentration of the working solutions (C_1_–C_35_) was 30 droplets. For each droplet, the spatial and temporal parameters of the deposition structures were measured in five cross-sectional regions.

Statistical processing and the plotting of all approximating curves were performed using the Origin Pro 2024 program (OriginLab Corporation, Northampton, MA, USA). All graphs present experimental data in means ± SD format.

## 5. Conclusions

Our study involved a systematic phenomenology and microscopic description of the self-organization of the adsorption structures (outer and inner ring ensembles, spikes of various sizes, loop ensembles, fractal structures, and dendritic branches) formed in a drying droplet at various BSA concentrations in 0.9% NaCl solution from 0.05 to 35 mg/mL.

The main parameters (number, size, growth times, periods of cycles and phases of drying droplets, etc.) of patterns at different BSA concentrations were assessed. The differences in the formation kinetics of outer and inner rings ensembles were discussed.

The elemental composition of the substances forming various precipitate patterns and structures was shown and analyzed. It was experimentally shown that the rings consist of two layers: a layer of BSA particle aggregates and a layer of NaCl crystals. The individual BSA particle aggregates had characteristic dimensions of 200–240 nm, and their density was 8–10 particles/µm^2^.

The results of this study can be used in medical diagnostics as biomarkers. The characteristic patterns presented in the work can be a useful experimental basis for further studies of the impact of physicochemical factors on proteins and other biopolymers.

## Figures and Tables

**Table 1:** Elements of adsorption structures formed during the drying of a droplet of an aqueous BSA–0.9%NaCl solution.

№	Structural Element	C_BSA_ < C_5_	C_5_ < C_BSA_ < C_20_	C_BSA_> C_20_
**1**	Outer rings	+		
**2**	Monocrystal surrounded by concentric rings	+		
**3**	Unidirectional polycrystal (spike)	+		
**4**	Rim of BSA dendrites	+	+	
**5**	Four-ray crystal surrounded by concentric rings		+	
**6**	Four-ray crystal with second- and third-order fractal-like structures		+	
**7**	Protein ring		+	+
**8**	Multi-ray polycrystal of fractal-like structure			+
**9**	Radially symmetric branched formations—dendrites extending uniformly from a single center in all directions			+

**Table 2:** Elemental composition of sedimentary structures in different local point of dried drop.

	**Weight Content, %**							**Weight Content Ratio**	
	**C**	**N**	**O**	**Si**	**Na**	**Cl**	**Other**	${\frac{1}{G}}_{W}$	$G_{W}$
P.c.	0	0	54.12	30.11	6.93	0.82	8.02	-	0
point 1	12.27	3.33	11.77	8.68	27.65	35.01	1.29	4.02	0.25
point 2	10.32	6.81	36.32	33.98	3.60	0.37	8.6	0.23	4.35
point 3	15.63	8.71	23.01	37.26	4.15	1.58	9.67	0.24	4.25
point 4	13.17	8.78	28.78	35.08	3.35	1.62	9.21	0.23	4.42
point 5	54.08	19.32	12.08	2.19	4.87	6.50	0.97	0.15	6.46
	**Atomic Content, %**							**Atomic Content Ratio**	
	**C**	**N**	**O**	**Si**	**Na**	**Cl**	**Other**	${\frac{1}{G}}_{A}$	$G_{A}$
P.c.	0	0	67.32	21.34	6.00	0.46	4.88	-	0
point 1	22.55	5.25	16.24	6.82	26.55	21.8	0.79	1.73	0.57
point 2	16.38	9.27	43.27	23.06	2.98	0.2	4.84	0.12	8,07
point 3	25.09	11.95	27.73	25.58	3.48	0.85	5.33	0.12	8.55
point 4	20.97	11.95	34.41	23.90	2.79	0.87	5.11	0.11	8.99
point 5	63.13	19.27	10.58	1.09	2.97	2.74	0.21	0.07	14.42

**Table 3:** Temporal relationships between the stages and phases of droplet drying.

$C_{BSA},\frac{mg}{mL}$	Complete Drying Cycle $t_{total},s$	Active Cycle $t_{a},c$	First Phase of Active Cycle $t_{2ph},s$	Second Phase of Active Cycles $t_{2ph},s$	$\frac{t_{a}}{t_{total}},\%$	$\frac{t_{1\;ph}}{t_{a}},\%$	$\frac{t_{1\;ph}}{t_{a}},\%$
$C_{0.05}$	172	20	14	6	12	70	30
$C_{0.25}$	224	30	22.5	7.5	13	75	25
$C_{0.5}$	210	20	15	5	10	78	22
$C_{1}$	122	11	7.5	3.5	9	68	32
$C_{5}$	200	7.5	-	7.5	3.75	-	100 ^1^

^1^ At a BSA concentration of 5 mg/mL, no outer rings are observed. Phase 1 of the active cycle is absent, and Phase 2 occupies the entire duration of the active cycle. The formation of inner rings occurs in the middle of the process and concludes somewhat before the droplet has completely dried. After this, the droplet continues to be filled with crystals—spikes of various shapes.

## Data Availability

The original contributions presented in the study are included in the article/Supplementary Materials. Further inquiries can be directed to the corresponding authors.

## Supplementary Materials

The following supporting information can be downloaded at: https://www.mdpi.com/article/10.3390/ijms27115060/s1.
